# Progress in Methanol Steam Reforming Modelling via Membrane Reactors Technology

**DOI:** 10.3390/membranes8030065

**Published:** 2018-08-17

**Authors:** Adolfo Iulianelli, Kamran Ghasemzadeh, Angelo Basile

**Affiliations:** 1 ITM-CNR, Via P. Bucci Cubo 17/C, University of Calabria, 87036 Rende, CS, Italy; a.basile@itm.cnr.it; 2 Department of Chemical Engineering, Urmia University of Technology, Urmia 57166-93187, Iran; kamran.ghasemzadeh@uut.ac.ir

**Keywords:** methanol steam reforming, hydrogen production, modelling, membrane reactors

## Abstract

Hydrogen has attracted growing attention for various uses, and, particularly, for polymer electrolyte membrane fuel cells (PEMFCs) supply. However, PEMFCs need high grade hydrogen, which is difficult in storing and transportation. To solve these issues, hydrogen generation from alcohols and hydrocarbons steam reforming reaction has gained great consideration. Among the various renewable fuels, methanol is an interesting hydrogen source because at room temperature it is liquid, and then, easy to handle and to store. Furthermore, it shows a relatively high H/C ratio and low reforming temperature, ranging from 200 to 300 °C. In the field of hydrogen generation from methanol steam reforming reaction, a consistent literature is noticeable. Despite various reviews that are more devoted to describe from an experimental point of view the state of the art about methanol steam reforming reaction carried in conventional and membrane reactors, this work describes the progress in the last two decades about the modelling studies on the same reaction in membrane reactors.

## 1. Introduction

In the last two decades, a growing interest towards the environment protection was noticed and more attention was gained by the research on renewable, alternative, and clean energy sources. In particular, fuel cells (FCs) were proposed as a viable option to the conventional processes for power production and to limit the greenhouse gases (GHGs) as well. Among various kinds of FCs, the polymer electrolyte membrane fuel cells (PEMFCs) are supplied by high grade hydrogen, which is industrially produced as a hydrogen-rich stream mainly via steam reforming of natural gas in fixed bed reformers (FBRs) [1]. Consequently, the hydrogen stream that is useful for PEMFCs supplying needs purification after the reforming process, which is conventionally realized in second stage processes, such as water gas shift (WGS) reactors (constituted of a high temperature reactor followed by a second low temperature reactor), partial oxidation (PROX) reactor, and pressure swing adsorption (PSA) [2]. Hence, the conventional process realizes the hydrogen purification with high costs and the need of various devices. As alternative candidates to the aforementioned conventional systems, inorganic membrane reactors (MRs) received much attention in the last 30 years because they combine the reforming reaction for hydrogen generation and its separation/purification in a single device [3,4]. In detail, among several inorganic membrane materials to be used in MR applications, a consistent literature was devoted on the development and utilization of palladium and/or palladium-alloyed membranes, which played a relevant role in this specific scientific field since palladium possesses the singular peculiarity of high hydrogen solubility and perm-selectivity with respect to all of the other gases [5,6,7,8,9,10,11].

In the meanwhile, according to the scientific literature, the methanol steam reforming (MSR) reaction has been studied a lot from an experimental point of view as attractive and promising process for generating hydrogen also in combination with inorganic MRs [12,13,14,15,16,17,18,19,20,21,22,23,24,25,26,27,28,29].

The whole MSR process can be described by the reactions reported in the following:

CH_3_OH + H_2_O = CO_2_ + 3H_2_   ΔH°_8K_= +49.7 (kJ/mol) (1)

CO + H_2_O = CO_2_ + H_2_   ΔH°_298K_ = −41.2 (kJ/mol) (2)

**Figure 1:**
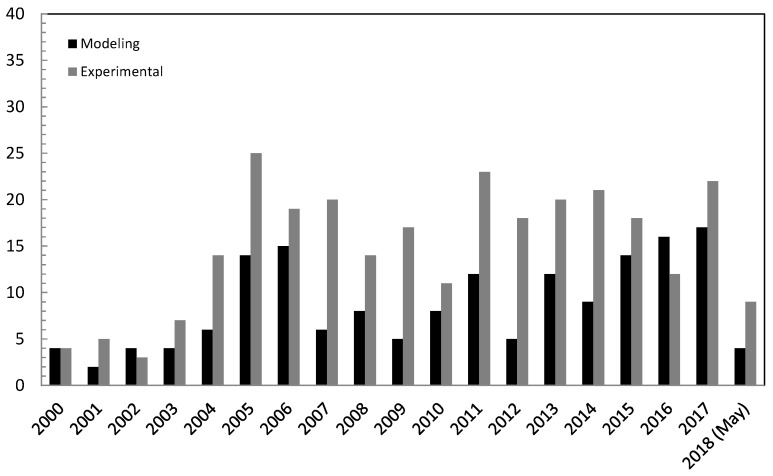
Number of scientific publication (modelling and experimental) versus year about methanol steam reforming (MSR) reaction in both fixed bed reformers (FBRs) and membrane reactors (MRs) (Scopus database: www.scopus.com).

CH_3_OH = CO + 2H_2_  ΔH°_298K_ = +90.7 (kJ/mol) (3)

where reaction (1) represents MSR, reaction (2) is the WGS, and reaction (3) is the methanol decomposition. As stated above, hydrogen production from MSR process has been extensively studied in both FBRs and MRs, as reflected by the number of publications per year reported in Figure 1.

Regarding the oscillation of the number of works per year for both modelling and experimental approaches, we believe that an experimental study may offer more opportunities for an original contribution due to new catalysts utilization and integration in a MR, new membranes tested, and so on. Otherwise, as also reported in Section 3, most of the simulations about the MSR reaction in MRs are one-dimensional (1-D) based, assuming full H_2_ perm-selectivity for the palladium membranes. This—in our opinion—limited the number of original contributions in this field and only recently the development of simulations using two-dimensional (2-D) and three-dimensional (3-D) models made an increase of new and original works about modelling of MSR reaction in MRs possible, as confirmed by a higher number of modelling papers than the experimental ones in 2016.

**Figure 2:**
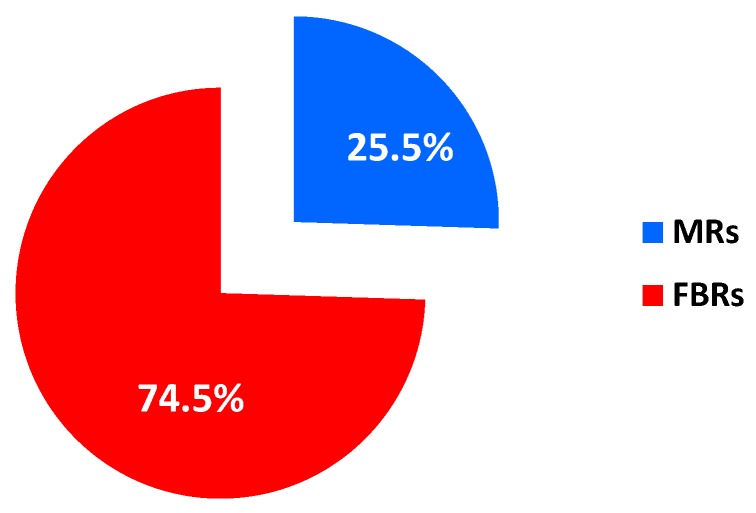
Percentage distribution about the modelling of MSR reaction involving FBRs and MRs (Scopus database: www.scopus.com).

Among the publications devoted to analyse MSR reaction from a modelling point of view, Figure 2 shows the percentage distribution between the studies involving FBRs and MRs.

This work is aimed to briefly describe the inorganic MRs technology from a modelling point of view, giving details regarding to the most recent findings in the MR assisted hydrogen generation from MSR reaction.

## 2. Inorganic Membrane Reactor Technology

Today, MRs represent a mature technology and a relevant progress was done in the last years to propose them as a valid alternative technology to the conventional systems. This is clearly evident in the field of hydrogen generation where the application of MRs successfully follows the principles of Process Intensification Strategy (PIS), being more attractive than the conventional systems concerning modularity and reduced costs [3,4,30,31].

**Figure 3:**
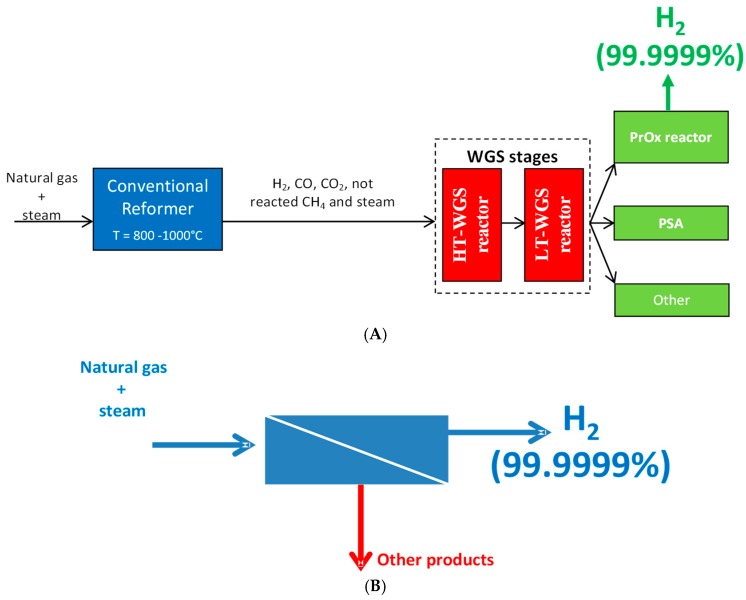
High grade hydrogen generation performed in: (**A**) a multi-stages conventional process; and (**B**) a MR housing a H_2_ perm-selective membrane.

As an example of PIS pursued by MRs, the production of high grade hydrogen from natural gas steam reforming reaction (the first source of hydrogen production at industrial scale [30]) is conventionally realized by means of a multi-stages process, Figure 3A; on the contrary, the utilization of a MR housing a hydrogen perm-selective membrane makes the combination of the reaction process for generating hydrogen and its purification in a single device without needing further hydrogen separation stages possible, Figure 3B.

The MRs can be categorized in fluidized and packed bed, while two distinct design solutions are possible: planar or tubular configuration. Apart from the experimental investigations about MRs, the mathematical modelling of a MR plays an essential role in process engineering. Indeed, the reactor modelling is useful to predict the behaviour of the system in dynamic/steady state conditions, optimizing the operating parameters via a suitable optimizing algorithm. This approach may be realized prior to running such an experimental test to avoid a longer operating time and efforts in the experimental campaign.

Important parameters of a hydrogen perm-selective membrane are the permeability and selectivity, responsible of the performance enhancement of a catalytic reaction. It is worth of noting that, generally, a membrane may act as an extractor, facilitating the selective removal of one of the products (i.e., hydrogen) from the reaction side toward the permeate side, shifting the reaction to the chosen direction, according to the Le Chatelier principle. Furthermore, the MRs adoption allows for the enhancement of the reaction conversion, reducing the by-product formation and lowering the energy requirements, driving consequently to a much flexible process [4].

Commonly, a packed bed MR allocates the catalyst in the tube/shell side of the membrane, whereas part of the hydrogen produced in the reaction side permeates through the membrane and is recovered in the shell/tube side. In this case, the permeation driving force is represented by the hydrogen partial pressure difference across the membrane [4,32].

On the contrary, a fluidized bed MR utilization presents different benefits over the packed bed modality, such as: improved heat and mass transfer and elimination of external mass-transfer resistances [33]. Nevertheless, the fluidized bed MRs show difficulties in the reactor manufacturing and catalyst erosion [34].

### 2.1. Palladium Membranes 

Many publications were addressed at the subject of hydrogen perm-selective membranes and much attention was received by metallic materials, such as Pd, Pd-alloy, Ti, Va, Mb, etc. [35,36,37,38,39,40,41]. Among them, palladium played a relevant role in this field and most of the literature is focused on this metal as a membrane material.

It is well known that the hydrogen permeation through the Pd-based membranes follows the so-called “solution/diffusion” mechanism, and, in the case of relatively low pressure, its rate-limiting step is assumed to be the diffusion [41]. The solution-diffusion mechanism may be expressed by six steps, as reported below:

1. hydrogen molecules adsorption from the membrane;
2. dissociation of hydrogen molecules on the membrane surface;
3. reversible dissociative chemisorption of atomic hydrogen;
4. reversible dissolution of atomic hydrogen in the metal lattice of the membrane;
5. diffusion into the metal of atomic hydrogen proceeds from the higher hydrogen pressure to the lower hydrogen membrane side;
6. desorption of re-combined atomic hydrogen into molecular form.

Hence, for a Pd-based membrane the hydrogen permeating flux may be expressed by the Equation (4):

J_H_2__ = Pe_H_2__ (p^n^_H_2,retentate__ − p^n^_H_2,permeate__)/δ (4)

where J_H_2__ is the hydrogen flux permeating through the membrane, Pe_H_2__ the hydrogen permeability, δ the membrane thickness, p_H_2,retentate__ and p_H_2,permeate__ the hydrogen partial pressures in the retentate and permeate zones, respectively, and n (varying between 0.5–1) is the dependence factor of the hydrogen flux on the hydrogen partial pressure. In the case of dense Pd-based membranes having thickness higher than 5 µm, Equation (4) becomes Sieverts-Fick’s law (5):

J_H__2,Sieverts-Fick__,_ = Pe_H_2__ · (p^0.5^_H_2,retentate__ − p^0.5^_H_2,permeate__)/δ (5)

Housing a Pd-based membrane in a MR, the enhancement of the reaction conversion with respect to the equivalent conventional reactor is promoted by the so-called “shift effect” [41]. Indeed, the hydrogen perm-selectivity characteristics of Pd-based membranes allow for the enhancement of conversion due to the hydrogen removal from the reaction toward the shell side.

### 2.2. MRs Modelling

Mathematical modelling of a MR represents an essential approach in membrane process engineering. Basically, MRs modelling is useful to predict the behaviour of the system in dynamic/steady state modality and optimize the operating conditions by a suitable optimizing algorithm. It can be realized prior to running the tests for avoiding longer operating time and efforts during the experimental campaign. Commonly, the models for realizing simulations on a MR are subdivided into three groups, which are described in brief.

#### 2.2.1. White Box or Theoretical Models

They are developed by adopting physical and chemical principles that are based on the conservation laws of mass, energy, and momentum as well as kinetic and transport equations and taking into account the physical behaviours of the MR. These models are firstly validated by experimental data, and, then, are applicable within a wide range of operation, giving a full insight through the system. Meanwhile, they are able to develop and solve models requiring significant time, needing specific hardware possessing a high-level of computational capacity.

#### 2.2.2. Black Box or Empirical Models

These tools work on the basis of the experimental data fitting and an example of them is represented by the Artificial Neural Network Models [42]. Among the characteristics of these models utilization, it is worth of noting that they are easy to derive but not helpful outside the conditions that are set in the experimental data.

#### 2.2.3. Grey Box or Semi-Empirical Models

These theoretical models adopt some parameters, such as the coefficients for the reaction-kinetics rate, catalyst adsorption, etc., which are calculated using data fitting [43]. These tools provide a clear system understanding, combined to a good generalization over a wide range of data, needing lower computational efforts than other theoretical models.

#### 2.2.4. Further Reactor Modelling Categorization

A further approach in categorizing the MRs modelling is based on the heterogeneous or pseudo-homogenous assumptions, which are responsible for the complexity and accuracy of the model itself. In a heterogeneous model, both fluid and the catalyst particles are seen as two different phases and the balance equations are described for both phases. On the contrary, in a pseudo-homogeneous model, they are considered as a single pseudo-phase and the balances equations are used for only one phase.

Generally, in modelling a conventional reactor using a white box, basically one should apply the mass, energy, and momentum balances, combined to the specific kinetics and transport equations. As a result, a set of differential equations (ODEs or PDEs) are obtained, and, through a suitable solving method, such results as the concentration profiles of each component, temperature and pressure in the reactor are simulated. By using the same approach for a MR, further parameters must be taken into account such as the hydrogen permeating flux and the heat transport inside and outside the reactor. Being a MR constituted of a reaction and a permeation zone, a hydrogen generation reaction is performed in the reaction side, whereas part of the produced hydrogen is removed from the reaction toward the permeate side for permeation through the membrane. Hence, four different streams may be considered: (1) feed: the inlet stream of the reaction side; (2) retentate: the outlet of the reaction side; (3) sweep: the inlet of the permeation side; and, (4) permeate: the outlet of the permeation side. Consequently, the balance equations are applied for both the reaction and the permeation sides, by using an accurate hydrogen permeating flux to correctly evaluate the removal of hydrogen from the reaction side. Furthermore, based on the type of reaction and the process needs, the catalyst may be packed in the tube or shell sides. Two configurations are then possible for the MRs, co-current and counter current. In the co-current mode, the feed in reaction zone and the sweep flow are introduced in the same direction, whereas in a counter current mode they are flowing in opposite directions.

#### 2.2.5. Tubular MR Modelling

**Figure 4:**
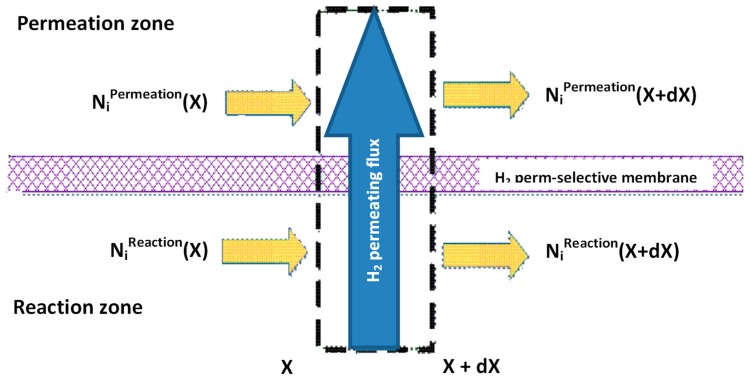
Schematic view of a mass transport in a MR.

Most of the modelling approaches about MRs belong to the tubular ones, because they represent the majority of the scientific studies present in the open literature and the most abundant in industrial applications. The mass balance for each MR side is related to a differential volume of length dX (Figure 4). Commonly, it is assumed that the compositions, temperature, and pressure vary along the axial direction without axial diffusion. In these conditions, the plug flow assumption is hence acceptable. 1-D models are the most common type for simulating a reaction process in MRs.

The simplification made by considering the properties variations in only one dimension allows for a simple solution of their derivations.

Due to the hydrogen permeation and heat transfer through the membrane, 2-D models also take into account radial concentrations and temperature gradients as well, including radial profiles and applying a different differential volume for two-dimensional modelling. Consequently, the radial diffusion is included in the balances, avoiding considering the term of hydrogen permeation in the balance equations, which is inserted as a boundary condition for both the permeation and reaction zones.

3-D modelling represent the most complex for simulating a MR, since they include the whole geometry and take into account the profiles in angular directions as well. Therefore, their applications are limited unless a non-symmetrical reactor is adopted.

In summary, four different modelling strategies may be applied for simulating a reaction process to generate H_2_ in MRs:

1. 1-D model, plug-flow;
2. 1-D model with axial diffusion;
3. 2-D model with axial and radial diffusion;
4. 3-D model with axial, radial and angular diffusion.

However, a deeper analysis about the main differences and peculiarities about the various models proposed in this work may be found in Alavi et al. [5].

##### Mass Balance

The mass balance is useful for calculating the concentration gradient in a MR. Generally, once a component balance is applied to a control volume of a dynamic reactor system, the principal terms of the mass balance equation are (1) the input and output flows of its component through the control volume, (2) the formation, permeation, and accumulation rates of its component in the control volume. The time dependent term is zero in steady state conditions and the reaction term only exists in the reaction zone modelling. The rate of hydrogen permeation depends on the type of membrane and on its permeation mechanism. It is positive for the permeation side and negative for the reaction side.

##### Concentration Polarization

The real hydrogen permeation driving force is represented by the Equation (4) and the exponent of the hydrogen partial pressure (*n*-value) may differ from 0.5, which is the typical coefficient describing the Sieverts-Fick law. As an example, during the permeation of hydrogen through a Pd-based membrane, a layer of non-permeating compounds accumulated on the membrane surface may involve a mass transfer resistance to the permeation process, representing the so-called concentration polarization effect, which may be responsible for a detrimental effect on the separation efficiency of the system. The fluidized bed MRs are able to minimize the concentration polarization effect [44]. Otherwise, the choice of smaller membrane diameter and bigger catalyst particles to avoid the pressure drop or the utilization of specific catalytic beds (structured catalysts) may facilitate the radial mixing properties, depressing the concentration polarization [45,46].

##### Energy Balance

For non-isothermal MRs, the energy balances are necessary to model their thermal performance. The prediction of the temperature variation inside a MR represents a crucial aspect because of the embrittlement phenomenon affecting the Pd-based membranes, which—as well known—takes place at relatively low temperatures (below 300 °C). Furthermore, in the case of composite Pd-based membranes applications, relatively high temperatures may determine different dilations of the two materials constituting the composite membrane, and then the membrane failure (especially in the case of ceramic substrates). Not less important is the control of the maximum operating temperature of the catalyst for ensuring that its activity cannot be affected.

##### Momentum Balance

In the case of the hydrogen permeation through the membrane and non-ideal flow pattern (not plug), the flow rate may be different and in small diameters the bed porosity that are close to the wall may be responsible for a non-uniform radial velocity profile, so that the momentum balance has to be included in the equations. Nevertheless, the inclusion of the momentum balance is not so common in the specialized literature, despite its advantages [5].

## 3. Modelling of MSR Reaction in MRs

Nowadays, to the best of our knowledge, there is not a consistent literature about numerical simulations of MSR reaction in MRs, also taking into account that this review analyzed the papers published after 2000 to illustrate the state of the art about modeling of MSR reaction in MRs in the last 20 years. Furthermore, most of the modeling papers about MSR in the aforementioned period were related to conventional reactors (please see Figure 2, in which it is indicated that ~75% of the papers on modeling of MSR reaction refers to conventional reactors), not in the scope of this review. Nevertheless, various studies focused on both conventional reactors and MRs [47,48,49,50,51,52,53,54,55,56,57,58,59,60,61,62] and most of them are based on 1-D model. As an exception, Fu and Wu [55] modeled MSR reaction in MRs while considering transient conditions and a couple of them used 2-D models to take into account the concentration polarization effect [47,53]. Most of the above referenced manuscripts refer to isothermal conditions, which are achieved only with an imposed cooling profile. Only a few of them deal with the study of non-isothermal conditions during MSR reaction [47,54,55].

Ideal gas behavior and plug flow are often assumed on both retentate and permeate sides, and heat gradients are neglected. Pressure drops are described by Ergun equation and palladium membranes are assumed to be defect free, hence showing a full hydrogen perm-selectivity, whereas mass and heat diffusions along the flow direction are usually neglected. Physical properties, such as heat capacity, gas density, and heat transfer coefficient are assumed constant with respect to the temperature variation.

In most of the aforementioned references, the pseudo-homogeneous formulation is adopted, and consequently, chemical reactions on the palladium surface are ignored. However, hydrogen removal from the reaction to the shell side is realistically considered by using experimental permeabilities. The finite difference method is used to solve the governing equations.

The prediction and optimization of the MRs performance during MSR reaction in terms of hydrogen yield and recovery and methanol conversion, as well constitute the final objective of the theoretical modelling by varying parameters such as H_2_O*/*CH_3_OH feed ratio, reaction temperature and pressure, sweep gas flow rate, etc.

**Figure 5:**
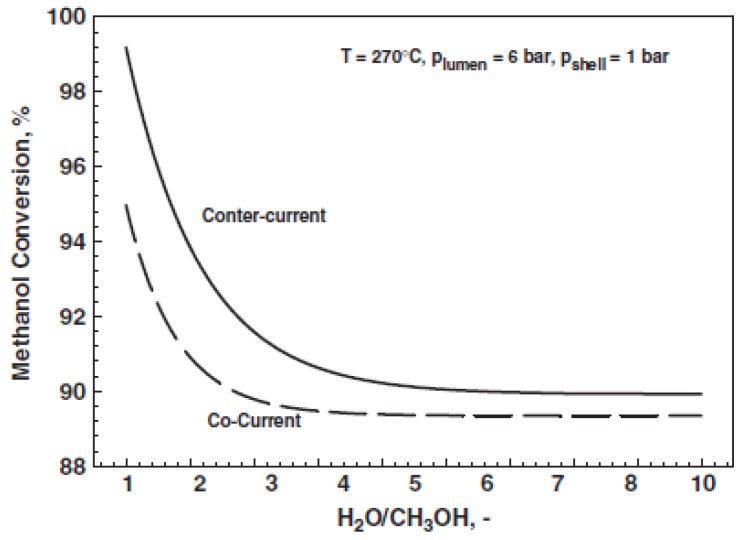
One-dimensional (1-D) modelling of MSR reaction in a Pd-based MR: methanol conversion versus feed molar ratio. With permission of reprint of Elsevier from Gallucci and Basile [52].

Gallucci and Basile [52] compared the performance of a MR with respect to the equivalent conventional reactor, while adopting a 1-D numerical model. A Runge-Kutta solution method was developed, including as reaction rate equations those of Peppley et al. [57], meanwhile comparing two different flow patterns (co-current and counter-current modality). These authors theoretically demonstrated that such parameters as reaction temperature, pressure, and feed molar ratio strongly affect positively the hydrogen production, while the adoption of the counter-current configuration is responsible of higher methanol conversions, Figure 5.

**Figure 6:**
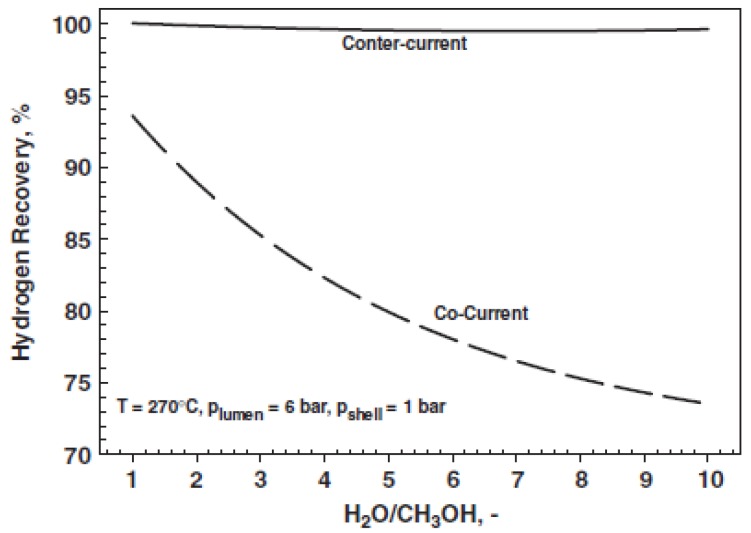
1-D modelling of MSR reaction in a Pd-based MR: hydrogen recovery versus feed molar ratio. With permission of reprint of Elsevier from Gallucci and Basile [52].

In detail, the model predicted that, with a H_2_O/MeOH ratio equal to 1/1 and at 270 °C and 6 bar, 99.5% methanol conversion may be reached in counter-current modality against 95% obtained with co-current mode adoption. Furthermore, the counter-current configuration acts positively on the hydrogen recovery, which is almost 100% in the whole range of feed ratio investigated. On the contrary, the co-current configuration is responsible for the decreasing trend of the hydrogen recovered in the permeate stream by increasing the feed ratio, Figure 6.

Nair and Harold [53] modeled MSR reaction in a Pd-Ag MR, in which the simulations were carried out in order to determine the conditions that are useful for maximizing the reactor productivity and the corresponding hydrogen utilization. In the simulations, the dusty gas model (DGM) was taken into account for pellet-scale diffusion and reaction.

Furthermore, the size of the catalyst particle was strictly considered for predicting the productivity. Indeed, Nair and Harold estimated that the maximum achievable productivity might be decreased by increasing the particle size. Furthermore, these authors identified both membrane thickness and surface to volume ratio as key design parameters. Consequently, they concluded that, for a given particle size, an optimum value of membrane surface/volume ratio allows for the maximum productivity to be reached. This conclusion is supported by the consideration that, at high membrane surface/volume ratio, insufficient catalyst is available to generate hydrogen and reduce the volumetric productivity. Otherwise, the effect of particle size on productivity becomes negligible. Concerning the role of membrane thickness, Nair and Harold stated that this is in strict correlation with the surface/volume ratio. Adopting thicker membranes a higher surface/volume ratio is achievable and smaller is the catalyst particles size. For instance, they simulated that a hydrogen productivity higher than 50 mol/m^3^·s is achievable at 260 °C and 10 bar, while using catalyst particles size smaller than 2 mm, membranes thickness lower than 10 μm, and membrane surface/volume ratio around 500 m^2^/m^3^.

**Figure 7:**
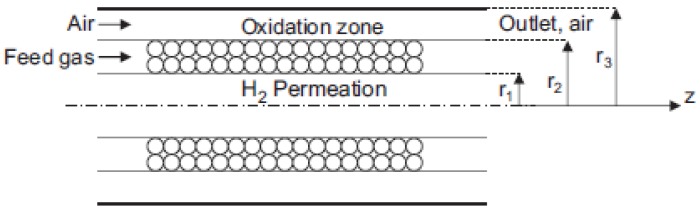
Conceptual scheme of a double-jacketed MR. With permission of reprint of Elsevier from Wu and Fu [54].

Fu and Wu [54,55] modeled the MSR reaction by using a double-jacketed Pd-based MR, while also considering a non-isothermal numerical model. The schematic diagram of the MR used by Wu and Wu [54] is shown in Figure 7, which illustrates that, from the external to the internal layer, three components are placed: the catalytic combustor, the reformer, and the permeator.

**Figure 8:**
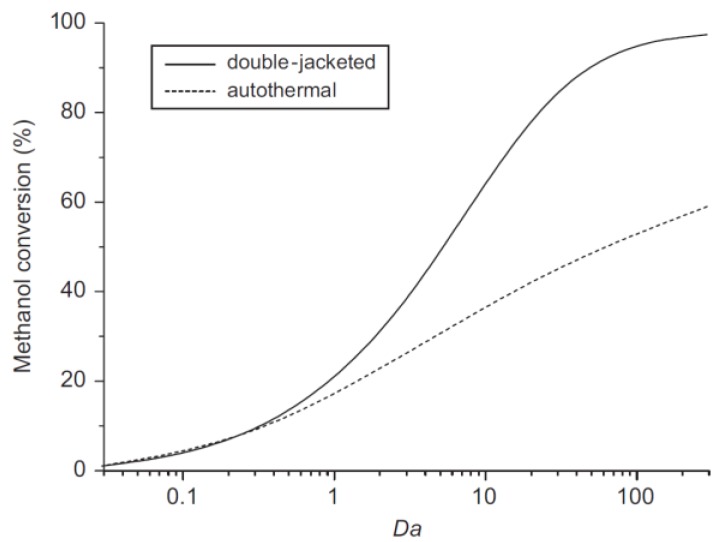
Methanol conversion *vs* Damköhler number: comparison between the double-jacketed MR and an equivalent autothermal conventional reactor. With permission of reprint of Elsevier from Wu and Fu [54].

The feed gas flows into the annular side (reformer) packed with a catalyst and the hydrogen permeates through the membrane. The unreacted gases flow into the oxidized zone and are mixed with air. As far as the modelling aspects are concerned, both mass and energy balances were evaluated simultaneously in annular and oxidation zones, as well as in the permeation side. The simulations pointed out that an increase of hydrogen volumetric flow rate in the permeation side produces an enhancement of hydrogen permeation rate across the membrane. Therefore, Fu and Wu established that an optimum ratio between radial (permeation) and axial (annulus) velocity is close to 10. Furthermore, for a specific Damköhler number, the higher the temperature the higher the hydrogen production, whereas methanol conversion and hydrogen recovery yield (defined as the percentage of pure hydrogen collected from the total hydrogen generation) are decreased. These authors also compared the performance of the aforementioned double-jacketed MR with an auto-thermal conventional reactor in terms of methanol conversion, hydrogen recovery, yield, and production rate. The simulations showed that, at the same operating conditions and for a definite reactor volume as well, a higher methanol conversion is reached by using the double-jacketed MR, Figure 8.

The numerical results evidenced this result for a Damköhler number higher than 1, while for a Damköhler number equal to 100, 95% methanol conversion is attained in the double jacketed MR with respect to 55% achievable adopting the auto-thermal conventional reactor. The double-jacketed MR was theoretically studied by the same authors also developing a transient model [55]. At the start-up, the temperatures of gases and catalysts as well as the consumption and species production were investigated in two MR conditions: (1) feed temperature higher than catalyst temperature, and (2) the reverse of condition (1). The simulations showed that condition (1) allows for obtaining higher methanol conversion and reactor temperature than condition (2). Moreover, during start-up the instability of species can be reduced with condition (1). The model analyzed also the MR response when a temporary extra hydrogen demand occurs under steady-state conditions. The latter could be satisfied by increasing the MR temperature from additional methanol oxidation or by increasing the inlet methanol.

**Figure 9:**
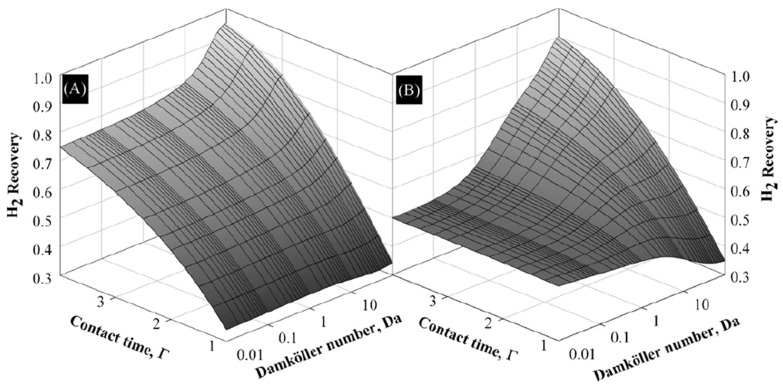
Simulation results of MSR reaction: Hydrogen recovery vs. contact time and Damköhler number: (**A**) carbon molecular sieve (CMS) MR, (**B**) Pd MR. With permission of reprint of Elsevier from Sà et al. [48].

MSR reaction was also theoretically studied by Mendes and co-workers [48], who compared the performance of Pd-based palladium and carbon molecular sieve (CMS) MRs in terms of methanol conversion and hydrogen recovery. Based on the experimental H_2_ permeabilities of CMS and Pd-based membranes from the open literature, the model predicted a similar methanol conversion being reached in both membrane reactors. This theoretical result could indicate that the permeation behaviors of the two membranes do not have any effect on the methanol consumption. On the contrary, the simulations evidenced that higher hydrogen recovery is achievable adopting a CMS-MR than a Pd-based MR, even though the latter may produce a pure hydrogen stream, Figure 9.

The hydrogen recovery is enhanced in both the MRs by increasing the Damköhler number. However, the Sà et al. [48] confirmed that the adoption of a Pd-based MR is more adequate if high grade hydrogen has to be produced because the simulations demonstrated that the H_2_/CO reaction selectivity is increased by using this reactor with respect to the CMS-MR.

**Figure 10:**
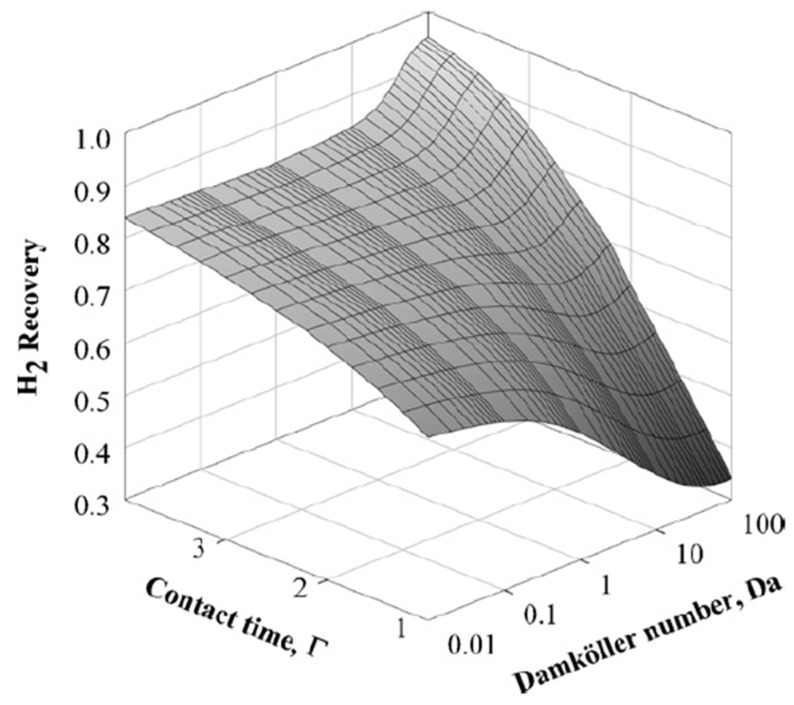
Simulation results of MSR reaction: Hydrogen recovery vs contact time and Damköhler number for a hybrid MR solution. With permission of reprint of Elsevier from Sà et al. [48].

As a further investigation, these authors analyzed a hybrid MR configuration consisting of a CMS membrane being positioned in series with a Pd-based membrane. This new configuration revealed some benefits, such as higher hydrogen recovery values when compared to MRs equipped with single membranes, Figure 10. 

Consequently, this hybrid MR solution allows for a reduced membrane area and higher feed flow rates than the Pd-MR, without a significant decrease in the performance. In another work, Sà et al. [14] modeled two different MRs for carrying out MSR reaction with the intent of obtaining high grade hydrogen for PEMFC supplying. 

**Figure 11:**
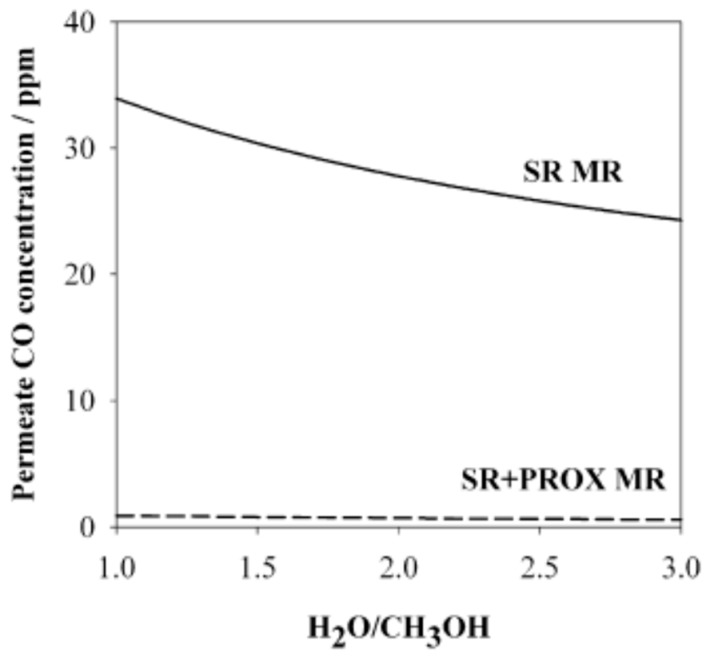
Simulation results of MSR reaction carried out in a MR and in a MR followed by a PROX reactor: Permeate CO concentration vs H_2_O/CH_3_OH feed molar ratio. With permission of reprint of Elsevier from Sà et al. [14].

**Figure 12:**
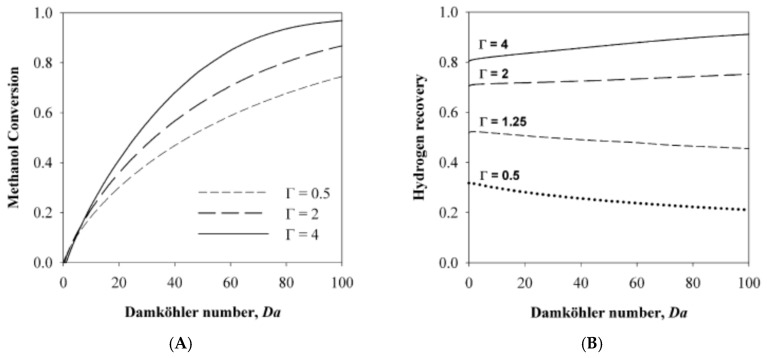
Simulation results of MSR reaction carried out in a MR followed by a PROX reactor: Methanol conversion (**A**) and hydrogen recovery (**B**) vs. Damköhler number at various contact time; With permission of reprint of Elsevier from Sà et al. [14].

The first system was based on a MR setup, whereas the second system was constituted by a PROX reactor in addition to a MR. In both cases, the governing equations were discretized with a finite volume method and the simulations showed the advantages due to the adoption of a PROX reactor. Indeed, the solution of the MR combined to the PROX reformer allowed for converting into CO_2_ (with a percentage below 20%) most of the CO contained in the MR permeate stream, showing a final CO content below 2 ppm, Figure 11. The simulations about this hybrid configuration showed that, at higher Damköhler number and contact time, both methanol conversion and hydrogen recovery are enhanced, Figure 12A,B, reaching the optimum at contact time values close to 2, where high methanol conversion (up to 85%) and hydrogen recovery (up to 75%) are >85% and 75%, respectively, meanwhile lowering the amount of CO_2_ to 10%.

**Figure 13:**
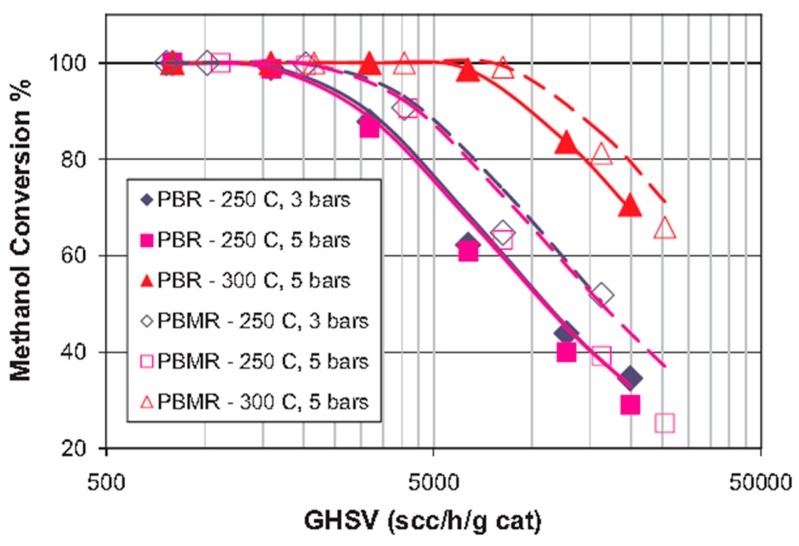
Simulation (lines) and experimental (symbols) results of MSR reaction carried out in a supported Pd-Ag MR and in a conventional reactor: Methanol conversions vs. gas hourly space velocity (GHSV) at different reaction temperature and pressure. With permission of reprint of Elsevier from Israni and Harold [47].

**Figure 14:**
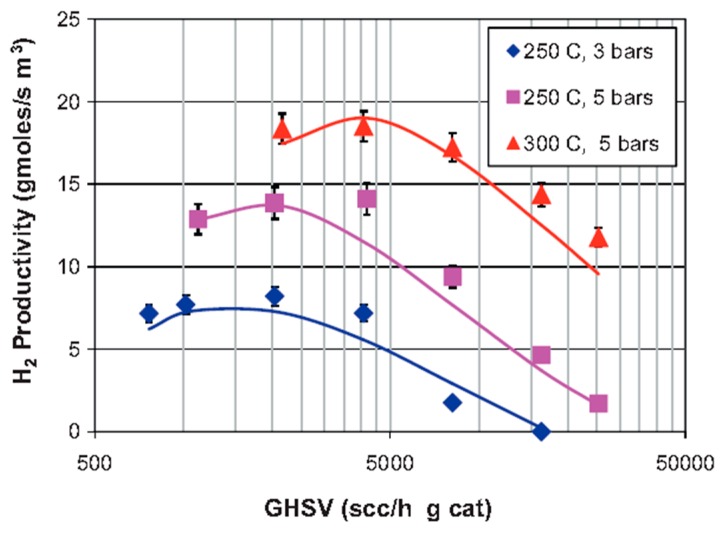
Simulation (lines) and experimental (symbols) results of MSR reaction carried out in a supported Pd-Ag MR and in a conventional reactor: H_2_ productivities vs GHSV at different reaction temperature and pressure. With permission of reprint of Elsevier from Israni and Harold [47].

Israni and Harold [47] proposed a 2-D and non-isothermal model to simulate MSR reaction in a MR housing a composite ~4 µm thick Pd-Ag membrane, when comparing the results with an equivalent conventional reactor. The authors included in the model the Maxwell equations for considering the molecular diffusion phenomena in the MR. Furthermore, a model of hydrogen flux inhibition due to the competitive adsorption of the primary MSR species was introduced, revealing a good agreement between the experimental and predicted concentrations. Figure 13 and Figure 14 show both experimental and modelling results concerning the influence of space velocity at different reaction temperature and pressure, on methanol conversion and hydrogen productivity, respectively. In both figures, the symbols represent the experimental results and the solid lines indicate the numerical results for the conventional reactor, while the dashed lines the numerical MR results. 

**Figure 15:**
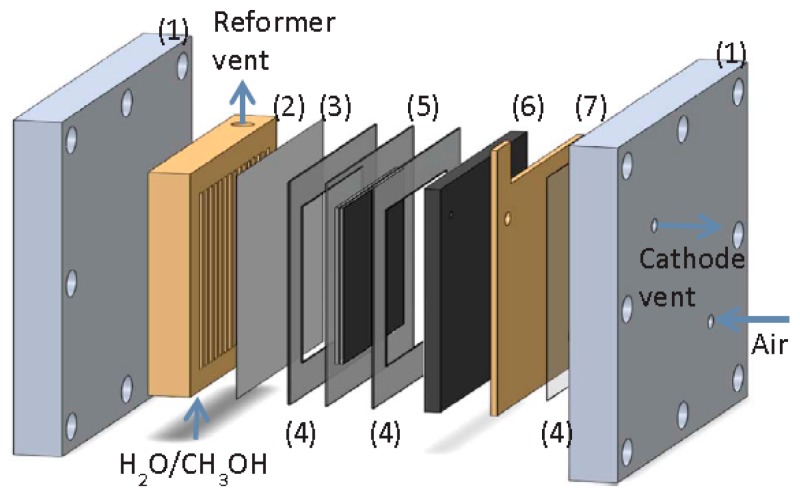
Schematic representation of the integrated system (MR + high temperature polymer electrolyte fuel cell (HT-PEMFC)): two metal endplates frames (1), gold coated reformer (2), Pd-Ag membrane (3), gasket (4), MEA (5), graphite composite bipolar plate (6) and current collector (7). With permission of reprint of Elsevier from Ribeirinha et al. [63].

More recently, Ribeirinha et al. [63] simulated an integrated process in which a MSR catalyst was packed into the anodic compartment of a high temperature polymer electrolyte fuel cell (HT-PEMFC), Figure 15. Here, both reforming and electrochemical reactions took place simultaneously. 

In addition, a Pd-Ag membrane, with a thickness of a few micrometers, was placed between the reforming catalyst and the membrane electrode assembly in order to avoid the contamination of the anode electro-catalyst by methanol. Hence, they used a 3-D non-isothermal model that was developed in Fluent (Ansys™) to simulate a packed bed MR combined to a HT-PEMFC in a single unit. The permeation characteristics of the membrane were taken by the experimental tests on a self-supported Pd-Ag membrane having a layer of 4 µm, which was produced by the magnetron sputtering technique and without pin-holes. At 200 °C, the H_2_/N_2_ perm-selectivity of the membrane was around 5800 and the hydrogen permeability around 2.9 × 10^−6^ mol·m·s^−1^·m^−2^·bar^−0.8^. 

**Figure 16:**
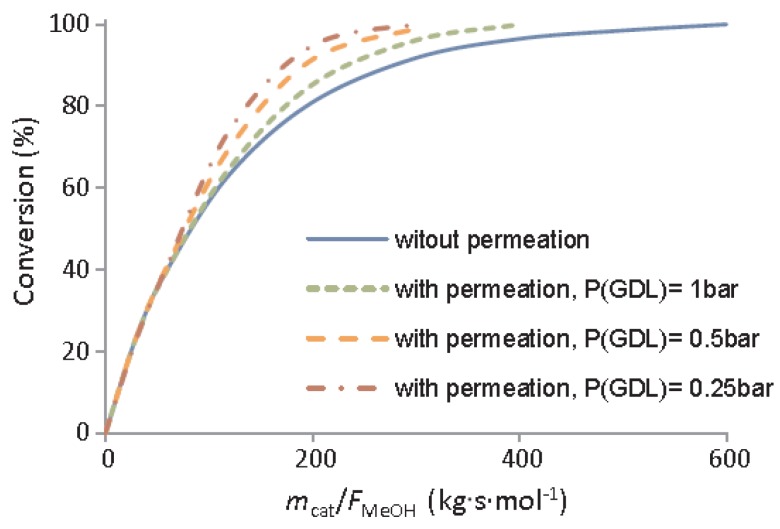
Simulated methanol conversion vs space-time in a Pd-Ag MR, at 200 °C, total retentate pressure of 3.0 bar. With permission of reprint of Elsevier from Ribeirinha et al. [63].

**Figure 17:**
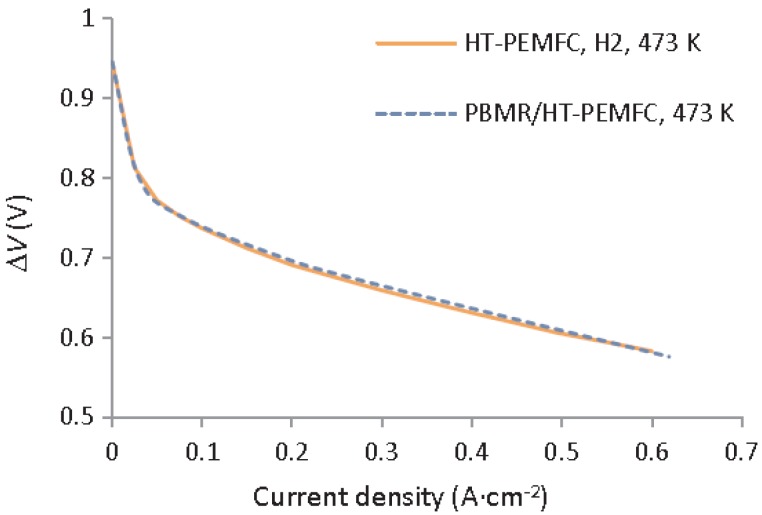
Simulations about the electric potential difference vs current density for a HT-PEMFC supplied by pure hydrogen and the integrated system combining a HT-PEMFC with a Pd-Ag MR operated at 200 °C. With permission of reprint of Elsevier from Ribeirinha et al. [63].

After proper validation of the model starting from the permeation behaviors of the membrane, the simulations pointed out high performance for the integrated system similar to the one that was obtained with a HT-PEMFC supplied by hydrogen, allowing for efficient heat integration between electrochemical and MSR reaction. In the MR simulations, Ribeirinha et al. [63] demonstrated the enhancement in the methanol conversion as the permeate pressure decreases, Figure 16. In this figure, methanol conversion is plotted against the space-time ratio in a Pd-Ag MR for different permeate pressures. To simulate a conventional reactor, the membrane was considered impermeable and—as shown—methanol conversion was always lower than the MR. In the combined configuration of the MR integrated with a HT-PEMFC, the hydrogen that was consumed by the electrochemical reaction may be responsible of the permeate pressure reduction below 1 bar, causing a faster hydrogen permeation through the membrane, acting as electrochemical hydrogen pumping. The success of the integrated system was confirmed by other simulations. These authors simulated the polarization curves by using a HT-PEMFC fed with pure hydrogen and the integrated system constituted of a HT-PEMFC coupled with the Pd-Ag MR-C at 200 °C, Figure 17. 

**Figure 18:**
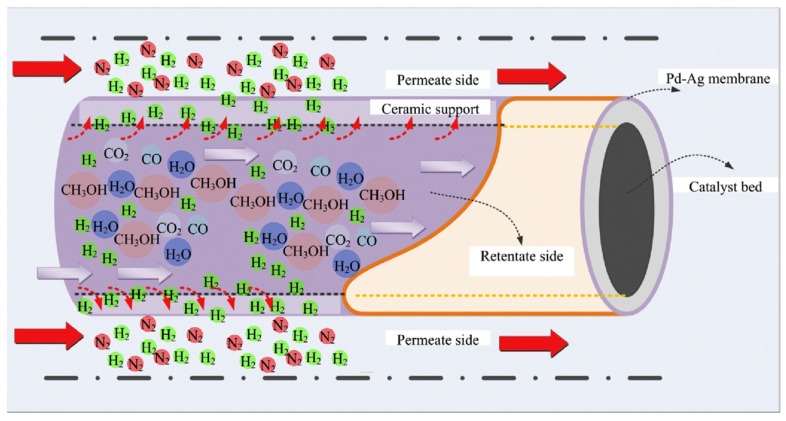
Scheme of the supported Pd-Ag MR. With permission of reprint of Elsevier from Saidi [64].

This figure shows how the results of the integrated system completely match those that were obtained with the HT-PEMFC supplied by pure hydrogen, indirectly confirming that the high methanol conversion and a very high hydrogen permeability of the Pd-Ag membrane cover the hydrogen consumption request for the HT-PEMFC supplying. Saidi [64] developed a comprehensive 2-D non-isothermal model to simulate the performance of MSR reaction in a supported Pd-Ag MR, evaluating the influence of different operating parameters, such as temperature, pressure, sweep ratio, and steam ratio on methanol conversion and hydrogen recovery, Figure 18.

**Figure 19:**
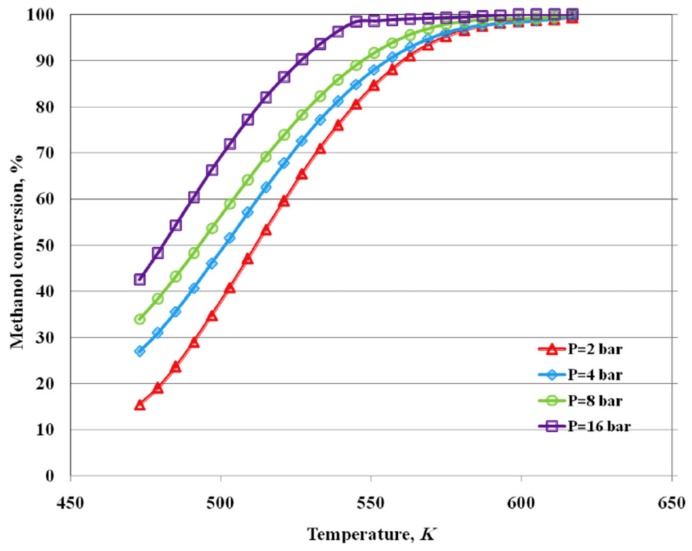
Simulation of MSR reaction in a supported Pd-Ag MR: methanol conversion vs temperature. With permission of reprint of Elsevier from Saidi [64].

The simulations evidenced that a temperature increase improves the kinetic catalytic properties and the hydrogen permeance through the supported membrane, resulting in higher methanol conversion and hydrogen production, Figure 19. This figure also shows that an increase of reaction pressure from 2 to 16 bar enhances methanol conversion more than 30%. This result points out that the role of the Pd-Ag membrane is crucial, because, by increasing the operating pressure, the hydrogen permeation driving force is intensified, determining a higher hydrogen removal with a consequent shift effect on MSR reaction toward further products formation and methanol conversion improvement. According to several literature data, it is evident that the permeation effect overcomes the thermodynamic effect, which represents the detrimental influence that is operated by higher pressures on MSR reaction, which proceeds from reaction to products with an increase of the moles number.

**Figure 20:**
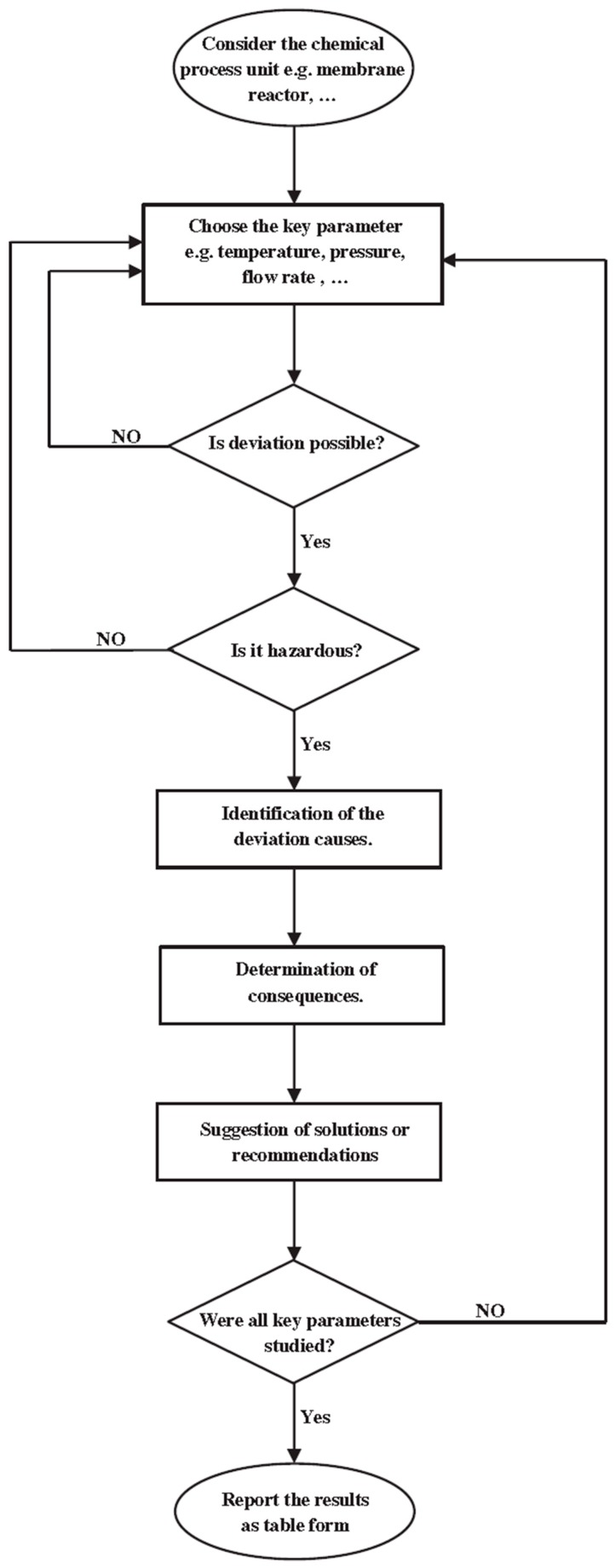
Scheme of the HAZOP approach. With permission of reprint of Elsevier from Ghasezmadeh et al. [65].

In conclusion, among several theoretical works [65,66,67], an original modelling study was realized by Ghasemzadeh et al. [65], who theoretically studied MSR reaction in a silica-based MR. These authors presented both a qualitative safety and a quantitative operating analysis of a silica-based MR adoption to perform MSR reaction for hydrogen generation. The safety analysis on the system was realized by using the HAZOP method (Figure 20). HAZOP (Hazard and Operability) analysis was developed in the early 1970s from a tentative approach to hazard identification for process plants to an almost universally accepted approach today, and a central technique of safety engineering. More details about HAZOP analysis may be found in Taylor [68].

Based on the HAZOP analysis approach, Ghasemzadeh et al. [65] made a comprehensive investigation about the most important operating parameters affecting the silica-based MR performance (quantitative analysis), which was realized by developing a 1-D isothermal model. The simulations evidenced that the reaction pressure and feed molar ratio have dual effects on the silica-based MR performance. On one hand, methanol conversion is decreased by increasing the reaction pressure from 1.5 to 4 bar, while over 4 bar, it is improved. On the other hand, the hydrogen recovery decreases by increasing the feed molar ratio from 1 to 5, while over 5 bar, it assumes a constant trend. Afterwards, the HAZOP analysis was carried out by using the analyzed operating variables as key parameters.

The safety assessment of MSR reaction performed in a silica-based MR was hence contained in different tables as check list. As an example, Table 1 reports the input-output table for deviations of the reaction temperature in the silica-based MR. However, the main goal of this modelling work was the nature itself of the recommendations resulted by the HAZOP analysis, constituting the solutions to avoid the economic and safety loss during MSR reaction in a silica-based MR.

## 4. Conclusions

The modelling approach represents a useful tool for simulating the physical and chemical phenomena in a MR, acquiring the adequate prior knowledge on the system by a reliable model. Indeed, the prior knowledge makes hence unnecessary the consumption of time and energy in the experiments. In this review, different modelling types were described when applied to MSR reaction carried out in MRs, giving a panoramic view on the recent advancements present in the open literature. Particular role was reserved to palladium membranes and much attention was dedicated from a theoretical point of view to the intensified process allowed by housing a Pd-based membrane in a MR. Most of the modelling studies present in literature involving MSR reaction and MRs include 1 or 2D models due to their easier derivation, even though more recently more complex 3D-modelling analyses were dedicated to a more accurate understanding toward the systems. Last but not least, this review also reported a new approach by combining the HAZOP analysis, useful for a safety evaluation of a silica-based MR used for generating hydrogen from MSR reaction, and a modelling analysis, useful for evaluating the main variables constituting the principal parameters of HAZOP algorithm. However, some issues still need to be better developed in the future of modeling approach about MSR reaction in MRs such as the membrane deactivation or the membrane performance variation in long-term uses, as main responsible effects on the global MR efficiency.

## List of Symbols and Acronyms

J_H_2__Hydrogen flux permeating through the membranenDependence factor of the hydrogen flux on the hydrogen partial pressurePe_H_2__Hydrogen permeabilityp_H_2,permeate__Hydrogen partial pressures in the permeate zonep_H_2,retentate__Hydrogen partial pressures in the retentate zoneδMembrane thicknessCMSCarbon molecular sieveFBRFixed bed reactorFCFuel cellGHGGreenhouse gasMRMembrane reactorMSRMethanol steam reformingODEOrdinary differential equationPDEPartial differential equationPEMFCProton exchange membrane fuel cellPISProcess intensification strategyPROXPreferential oxidationPSAPressure swing adsorptionWGSWater gas shift

## Figures and Tables

**Table 1:** Safety assessment of MSR reaction performed in a silica-based MR via HAZOP analysis: input-output table for deviations of the reaction temperature in the MR. With permission of reprint of Elsevier from Ghasezmadeh et al. [65].

Guide Words	Causes	Consequences	Recommensations
Less	1. Heater controller fails	Lower reaction rate	1. Check of the heater controller
	2. Lower temperature of feed reactants or HPLC pump fails	Lower hydrogen permeation	2. Check values and lines or HPLC pump
	3. Lower temperature of sweep gas	Lower hydrogen productivity	3. Check the sweep gas cylinder
	4. Isolation of MR set up fails	Lower hydrogen selectivity	4. Check of the isolation system
		Lower conversion	
		Lower hydrogen recovery	
		Condensation of vapors	
More	1. Heater controller fails	Thermal stress for the silica membrane	1. Check control system of heater
	2. Higher temperature of feed reactants or HPLC pump fails	Catalyst sintering	2. Check values and lines or HPLC pump
	3. Isolation of MR set up fails	Sealing of module fails	3. Check of the isolation system
		Formation hot spot in MR	
Tolerance	1. Heater controller fails	Defect formation on silica membrane	1. Check control system of heater
